# Near-infrared light-activated membrane fusion for cancer cell therapeutic applications†
†Electronic supplementary information (ESI) available. See DOI: 10.1039/d0sc00863j


**DOI:** 10.1039/d0sc00863j

**Published:** 2020-05-07

**Authors:** Fujian Huang, Ruilin Duan, Zhixin Zhou, Margarita Vázquez-González, Fan Xia, Itamar Willner

**Affiliations:** a Engineering Research Center of Nano-Geomaterials of Ministry of Education , Faculty of Materials Science and Chemistry , China University of Geosciences , Wuhan 430074 , China . Email: huangfj@cug.edu.cn ; Email: xiafan@cug.edu.cn; b Institute of Chemistry , Center for Nanoscience and Nanotechnology , The Hebrew University of Jerusalem , Jerusalem 91904 , Israel . Email: willnea@vms.huji.ac.il

## Abstract

The spatiotemporal stimulation of liposome–liposome or liposome–membrane fusion processes attracts growing interest as a means to mimic cell–cell interactions in nature and for using these processes for biomedical applications. We report the use of *o*-nitrobenzyl phosphate functionalized-cholesterol tethered nucleic acid-modified liposomes as functional photoresponsive units for inducing, by NIR-irradiation, spatiotemporal liposome–liposome or liposome–membrane fusion processes. The liposomes are loaded with upconversion nanoparticles (UCNPs) and their NIR irradiation (*λ* = 980 nm) yields luminescence at *λ* = 365 nm, providing a localized light-source to deprotect the *o*-nitrobenzyl phosphate groups and resulting in the fragmentation of the nucleic acid structures. In one system, the NIR-triggered fusion of two liposomes, L_1_ and L_2_, is exemplified. Liposome L_1_ is loaded with UCNPs and Tb^3+^ ions, and the liposome boundary is functionalized with a cholesterol-tethered, *o*-nitrobenzyl phosphate caged hairpin nucleic acid structure. Liposome L_2_ is loaded with 2,6-pyridinedicarboxylic acid, DPA, and its boundary is modified with a cholesterol-tethered nucleic acid, complementary to a part of the caged hairpin, associated with L_1_. NIR-irradiation of the L_1_/L_2_ mixture resulted in the photocleavage of the hairpin structure, associated with L_1_, and the resulting fragmented nucleic acid associated with L_1_ hybridized with the nucleic acid linked to L_2_, leading to the fusion of the two liposomes. The fusion process was followed by dynamic light scattering, and by monitoring the fluorescence of the Tb^3+^–DPA complex generated upon the fusion of the liposomes and their exchange of contents (fusion efficiency 30%). In a second system, the fusion of the liposomes L_1_, loaded with UCNPs and doxorubicin (DOX), with HeLa cancer cells functionalized with nucleic acid tethers, complementary to the hairpin units associated with the boundary of L_1_, and linked to the MUC-1 receptor sites associated with the HeLa cells, through a MUC-1 aptamer unit is exemplified. The effect of DOX-loaded L_1_/HeLa cell fusion on the cytotoxicity towards HeLa cells is addressed. The NIR UCNP-stimulated cleavage of the *o*-nitrobenzyl phosphate caged hairpin units associated with L_1_ leads to the fragmentation of the hairpin units and the resulting nucleic acid tethers hybridize with the nucleic acid-modified HeLa cells, resulting in the liposome–HeLa cell fusion and the release of DOX into the HeLa cells. Selective spatiotemporal cytotoxicity towards HeLa cells is demonstrated (*ca.* 40% cell killing within two days). The study presents a comprehensive stepwise set of experiments directed towards the development of NIR-driven liposome–liposome or liposome–membrane fusion processes.

## Abstract 2

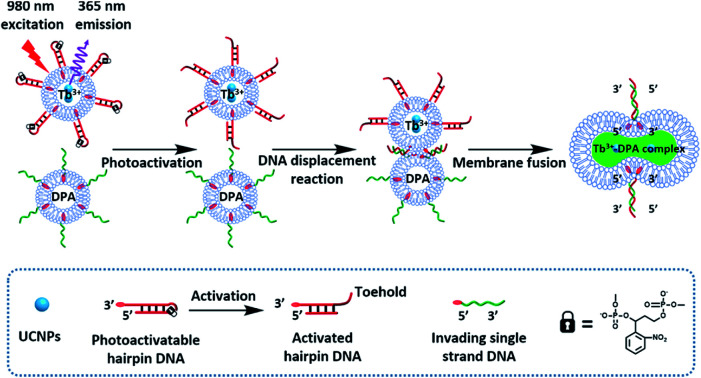
A NIR light activatable membrane fusion method was developed for cancer cell therapeutic applications.

## Introduction

The development of artificial cell-mimicking compartments, *e.g.*, artificial cells is a holy-grail challenge in the rapidly developing area of “systems chemistry”.^1^–^3^ Different self-organized micro/nanostructures such as liposomes,^4^–^7^ polymersomes,^8^–^10^ aqueous microdroplets,^11^ dendrosomes,^12^,^13^ and microcapsules^14^,^15^ were introduced as biomimetic cell structures. By the incorporation of loads into cell-mimicking compartments, cell-like functions such as controlled catalysis and biocatalysis in these compartments were demonstrated.^16^,^17^ In addition, the incorporation of stimuli-responsive elements into the boundaries of “artificial cells” allowed the triggered release of loads across the cell-like interfaces.^18^,^19^ Different triggers, such as temperature,^20^ light,^21^,^22^ pH,^23^–^25^ redox reagents,^26^ magnetic fields^27^ and ultrasound,^28^ were applied to induce the release of the loads from artificial cells. The inter-cell interactions and, particularly, cell–cell fusion represent important processes in biological systems such as neurotransmission^29^,^30^ exo- and endocytosis,^31^ signal transduction^32^–^35^ and viral infection.^36^,^37^ For example, soluble *N*-ethylmaleimide-sensitive-factor attachment receptors (SNAREs) represent a broad class of proteins and protein subunits that induce membrane fusion.^38^–^40^ Not surprisingly, substantial research efforts are directed towards the development of fusion pathways of cell-like compartments, *e.g.*, liposomes, as biomimetic model systems.^41^,^42^ In this context, the intrinsic recognition of complementary nucleic acids or peptide nucleic acids provides a versatile means to interconnect liposomes and induce liposome–liposome fusion.^43^–^45^ For example, lipidated or cholesterol-modified complementary nucleic acids incorporated into the boundaries of two different kinds of liposomes allowed the inter-hybridization of liposomes and their fusion.^46^–^49^ Nonetheless, these fusion processes were not triggered by auxiliary stimuli, thus eliminating spatiotemporal fusion events. The fusion efficiency of the liposomes was affected by the geometries and duplex lengths of the bridging nucleic acids.^50^–^52^ Also, the SNARE-mediated fusion of phospholipid vesicles was enhanced by lipidated nucleic acid tethers.^53^ In addition, the pH-induced triple-stranded assembly of lipidated peptides and the formation of ion-complexes of boronate ester bridges using appropriate lipidated ligands were demonstrated.^54^ Different applications of the fusion of liposomes were suggested and they include the detection of miRNAs by nucleic acid-mediated fusion of liposomes^55^ or the miRNA-induced inhibition of the nucleic acid-functionalized liposomes,^56^ and the barcoding of bioreactions through dictated fusion of vesicles.^33^,^57^,^58^ In addition, liposome–membrane fusion processes were suggested for drug release applications.^59^,^60^ While impressive progress in the understanding of the fusion process of liposomes was made, there are several difficulties underlying the processes. While interconnection of liposomes, *via* the different mechanisms, is obvious, the evaluation of the yield of the full fusion of the two liposomes that form an enlarged liposome, demonstrating the exchange of loads present in the two parent liposome compartments, is important. Different physical means to follow the interconnection and full fusion of liposomes, *e.g.*, dynamic light scattering (DLS),^61^,^62^ surface plasmon resonance (SPR),^63^,^64^ and photophysical methods to quantitatively analyze the fusion process by following the FRET or fluorescence properties of the contents in the fused structures, were introduced.^65^,^66^

The fusion of liposomes provides the basic principle for the fusion of liposomes with the cell membrane and the incorporation of loads into the cell cytoplasm. Indeed, nucleic acid-modified liposomes could be fused with lipidated- or cholesterol-functionalized cells, resulting in the delivery of the liposomal loads into the cells.^48^ Such liposome–cell fusion processes could be an indispensable pathway for nanomedicine by providing means to introduce drugs into cells and, particularly, anti-cancer drugs into cancer cells.^59^

The spatiotemporal control over the fusion of cells plays an important role in biological systems (selective and vectorial targeted fusion of cells in space, and time-dominated fusion of cell membranes). While substantial progress in the development of spontaneous fusion of liposome mixtures modified with complementary recognition elements was achieved, the examples of spatiotemporal control over the fusion of liposomes are scarce. The development of such systems would require the incorporation of: (i) a stimuli-responsive unit into the fusion element which enables the time- and space-triggered activation of the fusion process. (ii) A targeting unit coupled to the fusion element which dictates spatial, vectorial and selective fusion of the membranes. In fact, the development of a controlled spatiotemporal membrane fusion process for controlled and triggered release of drugs into target cells, could be important for cancer therapy.^48^ An impressive example for temporal control over membrane fusion has been demonstrated by the preparation of two oppositely loaded liposomes charged with coiled-coil polyethylene glycolated E and K SNARE peptides, where one of the peptide-modified liposomes was shielded with a lipidated photolabile *o*-nitrobenzyl ester polyethylene glycol chain.^67^ While the shielding of the peptide promoter unit prohibited fusion, the UV light-induced cleavage of the photoprotecting units and the sequestered removal of the polyethylene glycol shielding tethers stimulated the temporal fusion of the membranes. The system introduced a principle for temporal membrane fusion, but it lacks the spatial control of membrane fusion. In addition, the use of UV light to deprotect the shielding units is a major disadvantage for future medical applications.

The base sequence encoded in nucleic acids provides, however, a rich “tool-box” for designing spatiotemporal membrane fusion processes. Beyond the use of nucleic acid complementarity (duplex formation) as a membrane fusion principle, one may introduce into the fusing counter-parts stimuli-responsive triggering units and/or targeting units to yield spatiotemporal membrane fusion processes. This includes the possible application of sequence-specific aptamers that target liposomes to specific cell receptors,^68^,^69^ and the use of different switchable, signal-triggered reconfigurable nucleic acid structures, by using triggers such as photoisomerizable intercalators,^70^ triplex,^71^ metal-ions bridged duplex^72^ and redox agents-functionalized nucleic acids.^73^ One particular functionality that was previously used to activate nucleic acid structures includes *o*-nitrobenzyl phosphate photoprotecting groups.^74^ For example, photochemical patterned deprotection of the *o*-nitrobenzyl phosphate monolayer followed by the hybridization chain reaction was used to generate DNA branched polymer patterns on surfaces.^75^–^77^ Also, *o*-nitrobenzyl phosphate nucleic acid-based drug-loaded microcapsules were used as functional carriers for the light-induced release of drugs.^21^ In addition, *o*-nitrobenzyl phosphate-modified DNA probes were used as light-activated functional structures for the optical or electrochemical sensing of miRNAs.^78^

In the present study we introduce different *o*-nitrobenzyl phosphate-protected nucleic acids as functional units that induce liposome–liposome and liposome–cell fusion processes. Besides the characterization of the different membrane fusion configurations, we address the application of the fusion of drug (doxorubicin)-loaded photo-responsive liposomes with cancer cells as a means to stimulate targeted spatiotemporal cytotoxicity towards cancer cells. The main achievements of the present study include: (i) the photo-responsive liposomes are loaded with upconversion nanoparticles (UCNPs).^79^ This allows the light-induced deprotection of the photolabile protecting groups, and the fusion of the different membrane systems, by near IR irradiation (*λ* = 980 nm). The UCNPs yield a localized 365 nm light source for the deprotection processes. It should be noted that the conventional light-induced deprotection of the *o*-nitrobenzyl phosphate groups requires harmful UV light (*λ* = 365 nm) as the light source. The NIR irradiation of the UCNP-loaded liposomes provides a means to generate UV light locally without damaging neighboring normal cells. (ii) In addition, we integrate aptamers as functional units to target the liposome to specific membrane receptors, thereby leading to the spatiotemporal control over liposome–membrane fusion. It should be noted that previous studies have demonstrated the targeting of antibody-modified upconversion nanoparticle macrophage membranes^80^ and antibody-modified upconversion particles were applied to image tumor cells^81^ and to stimulate the photochemical generation of reactive oxygen species for photodynamic cancer cell therapy.^82^ Nonetheless, in all of these studies, upconversion nanoparticles were applied as fluorescent labels or intracellular catalysts yet not used as active membrane–membrane or membrane–cell fusion promoters. The present study introduces new principles to apply integrated upconversion nanoparticles in liposomes carrying drugs or optical labels as vehicles to actively induce membrane–membrane and membrane–cell fusion accompanied by the controlled release of imaging agents and drugs.

## Results and discussion

**Fig. 1:**
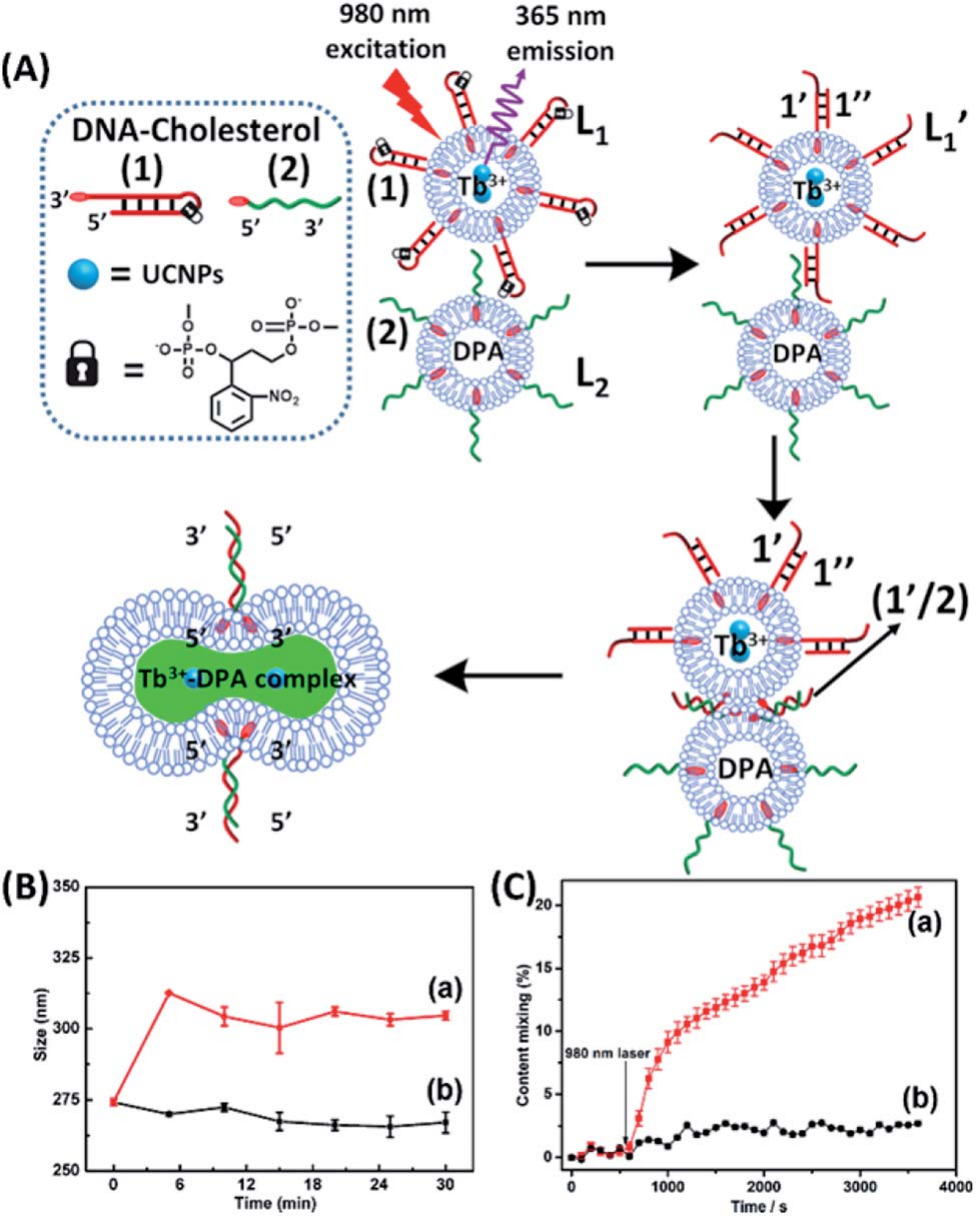
(A) Schematic of photochemically triggered fusion of liposomes L_1_ modified with *o*-nitrophenyl phosphate locked hairpin units (**1**) with liposomes L_2_ modified with nucleic acid (**2**). The liposome L_1_ is loaded with upconversion nanoparticles, UCNPs, and with Tb^3+^-ions. Liposomes L_2_ are loaded with the ligand DPA. NIR irradiation of the system, *λ* = 980 nm, leads to the UCNP-stimulated generation of the 365 nm internal light source that deprotects the *o*-nitrophenyl phosphate units and fragments the hairpin structure (**1′**/**1′′**). The (**2**)-functionalized liposome displaces the strand (**1′′**) resulting in the (**1′**/**2**) crosslinked liposome that leads to fusion. The fusion yields the fluorescent Tb^3+^–DPA complex that quantifies the full fusion efficiency. (B) Time-dependent size-changes of the liposome mixture L_1_/L_2_, evaluated by dynamic light-scattering upon: (a) the NIR-irradiation of the mixture of liposomes L_1_ and L_2_. (b) Non-irradiated mixture of L_1_ and L_2_ (error bars derived from *N* = 4 experiments). (C) Time-dependent percentage contents of the fused liposomes (or liposome fusion efficiency) corresponding to: (a) the NIR-irradiated mixture of liposomes L_1_ and L_2_ (liposome fusion efficiency was calculated according to the fluorescence increase corresponding to the Tb^3+^–DPA complex generated upon the full fusion of the two liposomes, see details in the Experimental section in the ESI†). (b) Non-irradiated mixture of the liposomes L_1_ and L_2_ (error bars derived from *N* = 3 experiments).

The near-infrared light-induced fusion of two liposomes is shown in Fig. 1. One type of liposomes, L_1_, is functionalized with 3′-cholesterol-tethered hairpin nucleic acid (**1**) units that are modified with *o*-nitrobenzylphosphate photoreactive bridging groups. The liposomes were loaded with upconversion nanoparticles (UCNPs) capable of converting near-infrared light (NIR) into UV light. In addition, the liposomes L_1_ were loaded with Tb^3+^ ions. The second type of liposomes L_2_ were functionalized at their boundaries with the 5′-cholesterol-modified nucleic acid (**2**). The (**2**)-functionalized liposomes were loaded with 2,6-pyridinedicarboxylic acid, DPA. The *o*-nitrobenzylphosphate bridging units stabilize the caged hairpin structure (**1**). The UV irradiation of the photoprotective groups, *λ* = 365 nm, leads to the cleavage of the *o*-nitrobenzyl phosphate units into *o*-nitrosobenzylphenone functionalities and to the fragmentation of the hairpin structure, resulting in the (**1′**/**1′′**)-modified liposome 
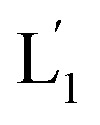
 (see Fig. S2, ESI†). As the liposomes L_1_ are loaded with the UCNPs, the NIR irradiation of the UCNPs, *λ* = 980 nm, leads to effective localized UV luminescence at 365 nm, resulting in the unlocking of the hairpin units associated with the liposomes L_1_ to 
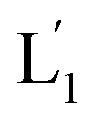
. As the (**1′**/**1′′**)-functionalized liposomes 
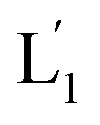
 include nucleic acid tethers complementary to the (**2**)-tethered liposomes, the formation of (**1′**/**2**) duplex bridged liposomes is anticipated to allow their fusion. The fusion process results in the exchange of the loads between the two liposome compartments, and thus, the fusion-guided exchange of the loads yields then the Tb^3+^–DPA fluorescent complex. Accordingly, the time-dependent fluorescence changes, in the L_1_/L_2_ mixture, upon the light induced uncaging of L_1_, and the time-dependent size changes (evaluated by light scattering) provide two physical means to probe the dynamics of fusion of the liposomes and to evaluate the yield of the full fusion of the liposome compartments leading to exchange of loads.

The photostimulated fusion process of the liposomes L_1_ and L_2_ was developed following several steps: (i) in the first (**1′**/**1′′**),-modified Tb^3+^-ion loaded liposomes and (**2**)-functionalized liposomes were prepared. Their mixture led to the fusion of the liposomes as evidenced by the time-dependent fluorescence changes and size changes of the fused liposomes, Fig. S1.† These experiments validated that the dynamic fusion process can be probed by the fluorescence change, upon formation of the Tb^3+^–DPA complexes and by the size changes accompanying the fusion process using dynamic light scattering experiments (see Fig. S1 and accompanying discussion†). (ii) In the second step, the photoresponsive (**1**)-functionalized liposomes L_1_ loaded with Tb^3+^, in the absence of UCNPs, were mixed with the (**2**)-modified liposomes. No fusion of the liposomes could be detected without UV light. The irradiation of the mixture with UV light, *λ* = 365 nm, induced the photodeprotection of the *o*-nitrobenzylphosphate units, the fragmentation of the hairpin units (**1**) and the formation of the (**1′**/**1′′**)-functionalized liposomes 
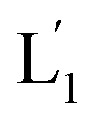
 that fused with liposomes L_2_ by the formation of inter-liposome (**1′**/**2**) duplexes, Fig. S2(A).† This is evident by the increase in the liposome sizes from *ca.* 250 nm diameter to *ca.* 300 nm sized liposomes, Fig. S2(B),† and by the time dependent increase in the fluorescence of the Tb^3+^–DPA complex generated upon the mixing of the loads associated with 
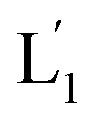
 and L_2_ upon fusion, Fig. S2(C).† (iii) In the third step, the integrated NIR-induced fusion of the liposomes, L_1_/L_2_, was examined, Fig. 1. In this system, the UCNPs were co-loaded with Tb^3+^ ions and the liposome mixture of L_1_ and L_2_ was irradiated with NIR light, *λ* = 980 nm. Under these conditions, the luminescence of the UCNPs yields localized UV light in the confined environment of L_1_. Under these conditions, the localized UV light cleaves the hairpin units (**1**), leading to the (**1′**)-functionalized liposomes, 
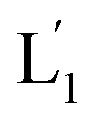
, that fuse with liposomes L_2_, Fig. 1(A). The time-dependent size changes of the liposomes upon irradiation of the liposome mixture with NIR light are presented in Fig. 1(B). The resulting fused liposomes reveal an average diameter of 310 nm while the non-irradiated liposomes show an unchanged diameter of *ca.* 260 nm. Also, Fig. 1(C) shows the time-dependent percentage content changes of the fused liposomes (or liposome fusion efficiency), upon NIR irradiation. The fluorescence of the Tb^3^–DPA complex increases and reaches a saturation value after *ca.* one hour, and the calculated liposome fusion efficiency is *ca.* 30%. It should be noted that this experiment was performed in the presence of 1 mM EDTA in the extravesicular solution to ensure that the resulting fluorescence does not originate from the light-induced lysis of the liposomes and the leakage of Tb^3+^ and DPA, upon irradiation of the system (see the Experimental section, ESI†). In fact, we find that the resulting fluorescence, upon irradiation of the system, is very similar, in the presence or absence of EDTA, in the extravesicular solution, implying that the fluorescence change was from the exchange of the components upon inter-fusion of the liposomes.

Control experiments confirm that no size changes or fluorescence changes in the liposome mixture occur in the absence of NIR irradiation. These results demonstrate that the fusion of the liposomes and exchange of the loads are, indeed, induced by the NIR activation of the fusion process. The yield of the full fusion, leading to exchange of the liposome loads, is comparable to the yields reported for other liposome–liposome fusion processes.^62^

In the next step, we examined the interactions of photoresponsive liposomes loaded with UCNPs and doxorubicin, DOX, an anti-cancer drug, with nucleic acid-functionalized cancer cells, *e.g.*, HeLa cells, with the vision that the fusion between the liposome and the cell could lead to a cytotoxic effect on the cancer cells. Such dynamically triggered therapy against cancer cells would need, however, several issues to be resolved: (i) the fusion between the photoresponsive liposomes and the cells, and the subsequent release of the loads associated with the liposomes should be demonstrated. (ii) An element for the selective fusion of the liposomes with the cancer cells should be introduced to reach selective cytotoxicity towards cancer cells. In the first steps described in Fig. S3 and S4, ESI,† several basic experiments confirming the fusion of the photoresponsive nucleic acid-functionalized liposome/HeLa mixtures were performed. In Fig. S3,† UV irradiation-triggered fusion of the liposomes L_3_ with HeLa cells, functionalized with cholesterol-modified *o*-nitrobenzylphosphate caged hairpin nucleic acid (**1**) was examined. The liposomes L_3_ were modified with nucleic acid (**2**) and FAM-functionalized nucleic acid (**4**) tethered at its 3′-end to cholesterol. The (**1**)-modified HeLa cells were irradiated with UV light (*λ* = 365 nm) to uncage (**1**) and induce the fusion between the liposomes and the cells. The confocal microscopy images confirmed the fusion between the liposomes L_3_ and the HeLa cells, Fig. S3(B)† and accompanying discussion. In Fig. S4,† NIR irradiation-triggered fusion of the (**1**)-hairpin and the FAM-tethered nucleic acid (**4**)-functionalized liposomes L_4_, loaded with UCNPs, with HeLa cells modified with the cholesterol-functionalized nucleic acid (**2**) was performed. The NIR-irradiation of the L_4_/HeLa cell mixture leads to the UCNP-stimulated cleavage of the hairpins associated with L_4_. The resulting (**1′**/**1′′**) duplexes are displaced by the tethers (**2**) linked to the HeLa cells, and the contacted liposome/HeLa cell assembly results in the fusion of the liposomes with HeLa cells and the release of UCNPs into the cytoplasm. The confocal microscopy images, Fig. S4(B),† confirm the interconnection between the liposomes L_4_ and the HeLa cells and the full fusion between the liposomes and the HeLa cells, resulting in the release of the luminescent UCNPs into the cells.

**Fig. 2:**
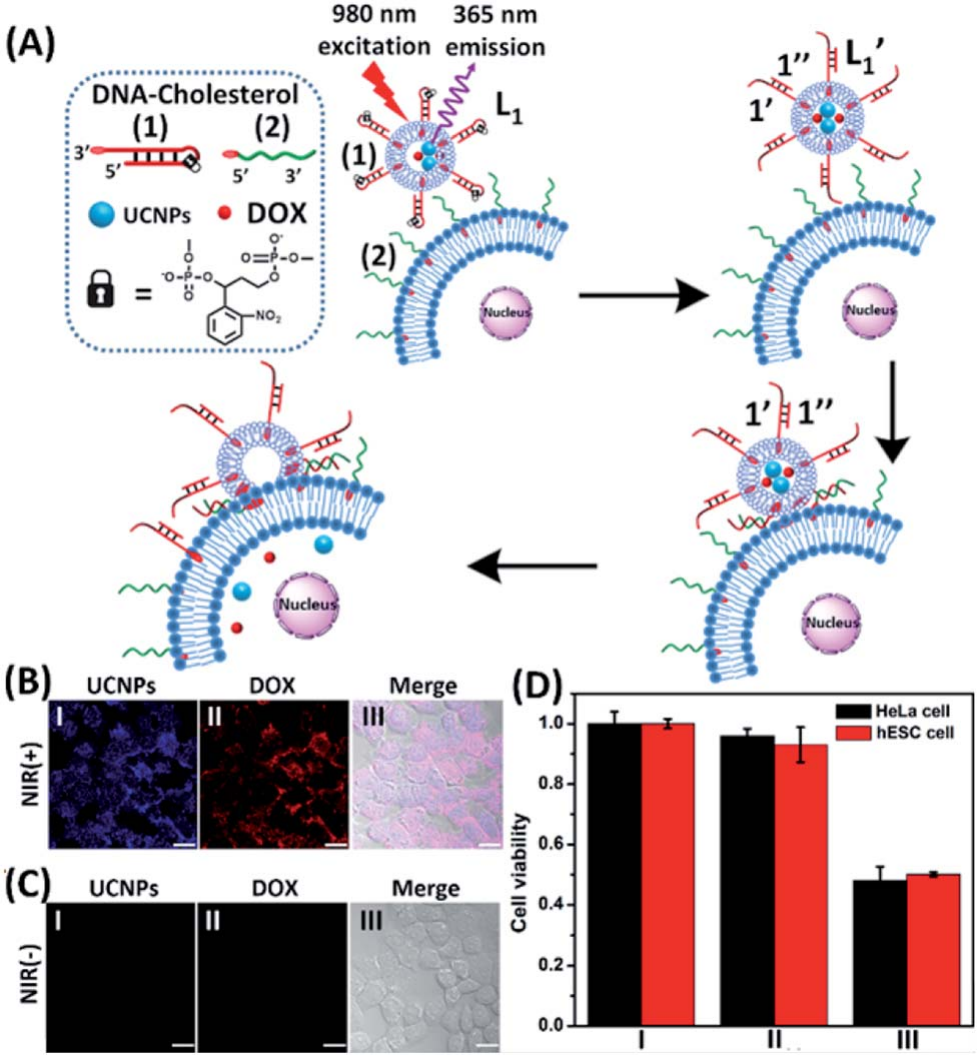
(A) Schematic of photochemically triggered fusion of the (**1**)-hairpin-functional liposomes L_1_, loaded with doxorubicin (DOX) and UCNPs, with HeLa cells modified with the cholesterol-functionalized nucleic acid (**2**). The NIR-irradiation of the L_1_/HeLa cell mixture leads to the UCNP-stimulated cleavage of the hairpins associated with L_1_. The resulting (**1′**/**1′′**) duplexes associated with 
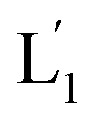
 are displaced by the tethers (**2**) linked to the HeLa cells and the (**1′**/**2**) contacted liposome/HeLa cell assembly results in the fusion and the release of DOX (and UCNPs) into the cytoplasm. (B) Confocal microscopy images corresponding to NIR-stimulated fusion of the (**1**)-modified liposome/HeLa cell mixture: panel I – blue channel fluorescence of the exchanged and released UCNPs into HeLa cells. Panel II – red channel fluorescence of the DOX released into the cells. Panel III – merged image demonstrating the overlap of fluorescence of the UCNPs and DOX in the fused cells (scale bar, 20 μm). (C) Confocal microscopy images corresponding to the non-irradiated (**1**)-modified liposome, L_1_/HeLa cell mixture: panel I and panel II show no fluorescence in the respective blue/red channels. Panel III – merged image demonstrating that no UCNPs and DOX were introduced into the HeLa cells (scale bar, 20 μm). From a set of *N* = 5 bright field images of the cells and the corresponding fluorescence responses of the overlapped confocal microscopy images, we estimate a fusion efficiency of DOX/UCNPs with the HeLa cells (or the normal hESC cells) of 85–90%. (D) Cytotoxicity of the UCNP and DOX loaded microcapsules L_1_ towards HeLa cells and hESC normal cells: entry I – non-treated cells; entry II – cells treated with liposomes loaded with UCNPs and DOX without irradiation. Entry III – cells subjected to NIR-irradiated liposomes loaded with UCNPs and DOX. Viability of the cells monitored after a time-interval of two days. Results show *ca.* 50% residual cell viabilities implying non-selective fusion of the DOX/UCNP-loaded liposomes L_1_ with the two types of cells: HeLa and hESC. Error bars derived from *N* = 3 experiments. It should be noted that the cytotoxicity experiments of photochemically fused DOX/UCNP-loaded liposomes into the HeLa cells (or hESC cells) were performed under conditions where cell cultures containing 7.3 × 10^6^ cells were subjected to 1.2 × 10^–9^ moles of DOX incorporated into the liposomes. Considering the fusion efficiency of the liposomes with the cells, the average loading of DOX in the cells corresponded to 1.5 × 10^–16^ moles per cell. The resulting 50% viability of the cells, after two days of interaction, could be attributed to insufficient loading of the cells with DOX. Thus, the cytotoxicity of the fused DOX/UCNPs could be enhanced by increasing the concentration of the liposomes, increasing the loading of DOX in the liposomes, and prolonging the time of interaction of the fused liposome/cell assemblies.

In the next step, we constructed a liposome/HeLa cell mixture, where the liposomes and HeLa cells were modified with appropriate nucleic acids, and the liposomes were loaded with UCNPs and doxorubicin (DOX), Fig. 2. The UCNP/DOX-loaded liposomes were functionalized with the photoresponsive *o*-nitrobenzyl phosphate caged hairpin (**1**) modified at its 3′-end with cholesterol. The HeLa cells were modified with the 5′-end cholesterol-functionalized strand, (**2**). Irradiation of the liposome/HeLa cell mixture with NIR light, *λ* = 980 nm, resulted in the 365 nm-stimulated cleavage of the hairpins (**1**), and the hybridization of the fragmented strand (**1′**) with the (**2**)-functionalized HeLa cells, leading to the fusion of the two compartments and to the incorporation of the UCNPs and DOX into the fused compartment, Fig. 2(A). The confocal fluorescence microscopy images of the NIR-irradiated cells are presented in Fig. 2(B). Panel I shows the blue channel fluorescence of the UCNPs incorporated into the HeLa cells, and panel II shows the red channel fluorescence of the cell-incorporated DOX. Panel III presents the merged fluorescence microscopy image of the cells. The microscopy images clearly indicate that the UCNPs and DOX are, indeed, released into the cell compartments as presented in Fig. 2(A). Fig. 2(C) shows the confocal microscopy images of the non-irradiated HeLa cell/liposome system. No incorporation of the UCNPs and DOX into the non-irradiated cells is observed, implying that the light-induced cleavage of the hairpin units associated with the liposomes is essential to allow the fusion of the liposomes with the cells, and the release of the UCNPs and DOX into the cells. Similar results are observed upon subjecting hESC normal cells to the UCNPs/DOX-loaded-(**1**)-modified liposomes, Fig. S5.† Thus, non-selective incorporation of the UCNPs and DOX into the HeLa and hESC cells occurs. Indeed, cytotoxicity tests, Fig. 2(D), reveal that the release of DOX into the cancer cells and the normal cells, results in comparable cytotoxicity towards the two types of cells. Entry II shows that no cytotoxic effect is observed in the non-irradiated mixture consisting of the UCNP-functionalized DOX-loaded liposomes and the HeLa or the hESC cells after two days of interaction. A significant cell death (*ca.* 50%) is observed upon the NIR irradiation of the mixture consisting of the UCNPs-functionalized, DOX-loaded liposomes and the (**2**)-modified HeLa cells or the hESC cells. In these experiments, the L_1_ liposome/HeLa cell mixture was irradiated with a NIR source for 10 minutes followed by the subsequent interaction of the mixture for two days, entry III. While effective cell death is observed, consistent with the NIR-stimulated fusion of the liposomes with the two cell membranes which results in the release of DOX into the cells, the process is not selective, and cytotoxicity toward the cancer cells and normal cells is observed.

**Fig. 3:**
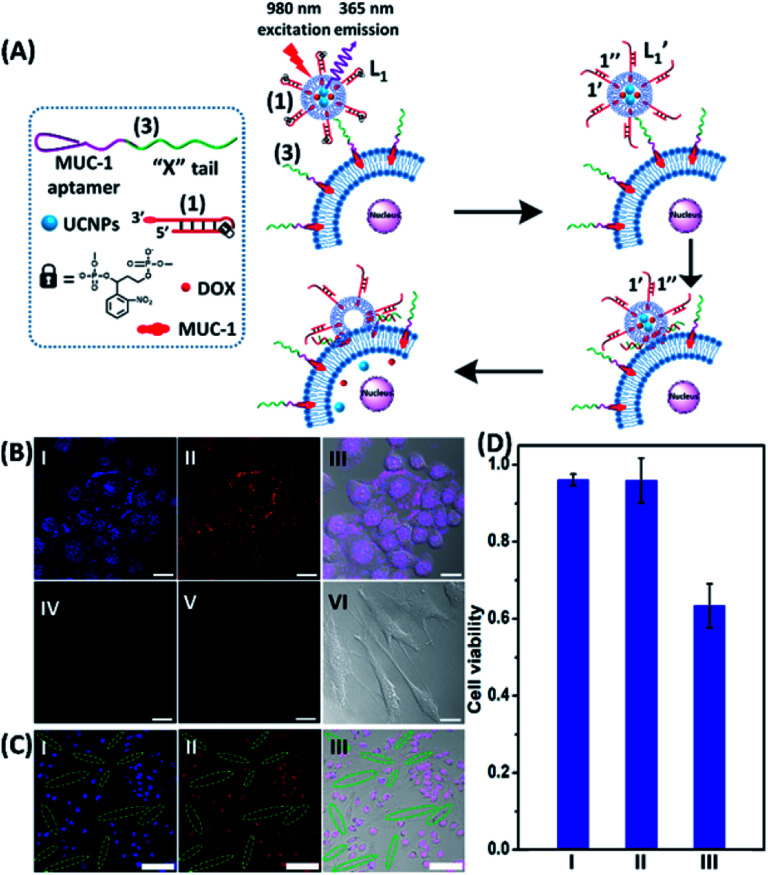
(A) Schematic of MUC aptamer-guided fusion of the DOX/UCNP loaded liposomes L_1_ with HeLa cells functionalized with strand (**3**) consisting of the MUC-1 aptamer coupled to the domain “X” composed of the sequence (**2**). The strand (**3**) binds to the MUC-1 ligand associated with the HeLa cells and the hybridization of the free tether “X” with the photo-fragmented duplex (**1′**/**1′′**) leads to the fusion of liposomes L_1_ with the respective cells and to the release of the UCNPs and DOX into the cells. (B) Confocal microscopy images corresponding to: panel I – blue channel of NIR-irradiated mixture of the UCNP/DOX-loaded liposomes L_1_ and the (**3**)-functionalized HeLa cells. Panel II – red channel fluorescence of DOX released into the cells. Panel III – merged image demonstrating the overlap of fluorescence of UCNPs/DOX released into the cells. Panel IV and panel V – blue and red channel, respectively, of the NIR-irradiated mixture of UCNPs/DOX-loaded liposomes, L_1_, and the hESC cells pretreated with (**3**). Panel VI – merged image of the blue/red channels and the bright field fluorescence image, demonstrating that no release of UCNPs or DOX into the hESC cells occurs (scale bar, 20 μm). (C) Confocal microscopy images of a mixture of HeLa cells and hESC normal cells treated with the UCNP/DOX-loaded liposomes, L_1_ and subjected to NIR-irradiation. The shapes of the cells and the fluorescence features of the two types of cells are used to differentiate the fusion behavior of the different-shaped cells. Panel I – blue channel demonstrating the fusion of the UCNPs only into HeLa cells (cell marked with dotted rims correspond to hESC cells). Panel II – red channel-DOX fluorescence restricted to the HeLa cells (no fluorescence in the elongated hESC cells, marked with dashed boundaries). Panel III – merged image demonstrating the selective fusion of UCNPs and DOX with the HeLa cells (scale bar, 100 μm). (D) Viability of the HeLa/hESC cells subjected to the UCNP/DOX-loaded liposomes L_1_: entry I – hESC cells treated with the NIR-irradiated UCNPs/DOX-loaded liposomes. Entry II – the HeLa cells subjected to UCNP-loaded liposomes L_1_, lacking the DOX load. The results indicate that despite the fusion of the liposomes with HeLa cells proceeding, the viability of the cells is preserved, demonstrating that the UCNPs are non-toxic towards the HeLa cells. Entry III – the HeLa cells treated with the UCNP/DOX-loaded liposomes L_1_ and subjected to NIR-irradiation. The results demonstrate that the fusion of the liposomes and the HeLa cells, and the release of DOX lead to selective cytotoxicity towards the HeLa cells.

To introduce cytotoxic selectivity towards the HeLa cells *via* the NIR-guided fusion of the UCNPs/DOX loaded liposomes with the cancer cells we apply specific aptamer–cancer cell receptor interactions as a means to guide the UCNPs/DOX to the cancer cells and specifically release the DOX drug in the targeted cancer cell, Fig. 3(A). Since the MUC-1 receptor is overexpressed in the HeLa cell boundaries and since an anti-MUC-1 aptamer is available, we designed a strand (**3**) which includes the MUC-1 aptamer sequence conjugated to the tether “X” that is complementary to the fragmented strand generated upon the NIR-irradiation of the (**1**)-functionalized UCNPs/DOX-loaded photoresponsive modified liposomes. Challenging the HeLa cells with the strand (**3**) leads to the binding of (**3**) to the HeLa cells through aptamer-MUC-1 complex formation. The NIR-irradiation of the mixture consisting of the (**3**)-functionalized HeLa cells and the UCNPs/DOX-loaded-(**1**)-functionalized liposomes leads to the light-induced cleavage of the photoresponsive *o*-nitrobenzyl phosphate caged hairpin (**1**). The fragmented tethers associated with the liposome boundaries hybridize with the domain X of strand (**3**), associated with the HeLa cells. This leads to the fusion of the liposomes with the cells and to the release of the UCNPs and DOX into the cells. Fig. 3(B), panels I–III show the confocal fluorescence microscopy and merged images of the NIR-irradiated mixture composed of the UCNPs/DOX-loaded-(**1**)-hairpin-modified liposomes and the (**3**)-modified HeLa cells. The HeLa cells, panel I, reveal the blue fluorescence of the UCNPs and the red fluorescence, panel II, associated with DOX. The dual fluorescence features of the fluorescent constituents, panel III, indicate that the fusion of the liposomes with the HeLa cells occurred, resulting in the release of the UCNPs (blue fluorescence) and DOX (red fluorescence) into the cancer cells. For comparison, the hESC normal cells were pretreated with the MUC-1 aptameric strand (**3**). As these cells lack the MUC-1 binding sites, no binding (or low binding) of (**3**) to these cells occurs. The fluorescence features of the NIR-irradiated mixture consisting of the UCNP/DOX-loaded-(**1**)-functionalized liposomes and the (**3**)-pretreated hESC normal cells are presented in Fig. 3(B), panels IV–VI. No blue fluorescence of the UCNPs or red fluorescence of DOX is detected in the cells indicating that no fusion of the liposomes occurred with the hESC cells. The merged image, panel VI shows, however, the elongated hESC cells that lack the UCNP/DOX loads. That is, the interaction of the cells with the MUC-1 aptamer-sequence (**3**) introduced selectivity for the incorporation of the UCNP/DOX-loaded-(**1**)-functionalized liposomes into HeLa cancer cells. Further support for the selective light-induced incorporation of the UCNP/DOX-loaded-(**1**)-modified liposome is observed upon treatment of a mixture of HeLa cells and hESC normal cells with the liposomes and imaging the permeation of the UCNPs/DOX into the two types of cells, exhibiting different shapes. Fig. 3(C), panels I–III show the fluorescence microscopy images and the merged image of the NIR-irradiated mixture of HeLa cells and hESC cells in the presence of the UCNP/DOX-loaded liposomes. The blue channel fluorescence of the UCNPs and the red channel fluorescence of DOX are observed in panels I and II, respectively, consistent with the release of the UCNPs/DOX upon fusion of the liposomes with the HeLa cells. The merged image, panel III, consists of the overlay of the fluorescence images and the bright field images of the mixture of cells. Clearly, the pink overlayed UCNP/DOX fluorescence is visible on the structures of HeLa cells, yet no fluorescence is observed on the hESC-elongated shaped cells (marked with dashed lines). These results are consistent with the selective fusion of the UCNP/DOX-loaded-(**1**)-modified liposomes with the HeLa cells. The selective fusion of the UCNP/DOX-loaded-(**1**)-modified liposomes, and the accompanying targeted release of the DOX drug into the HeLa cells suggest that the selective cytotoxicity towards the respective cells can be accomplished. Fig. 3(D) depicts the cytotoxicity experiments. In this study, in entry I, the hESC cells were treated with the MUC-1 aptamer and mixed with the UCNP/DOX-loaded-(**1**)-modified liposome, and the mixture was subjected to NIR irradiation. The viability of the cells after two days corresponded to >95%. This result is consistent with the lack of fusion and release of DOX into the hESC cells. In entry II, the MUC-1 aptamer functionalized HeLa cells were interacted with the UCNP-loaded-(**1**)-modified liposomes and subjected to NIR irradiation. These liposomes lack DOX, yet still allow the light-induced fusion of the liposomes with the HeLa cells and the release of the UCNPs into the cells. After two days, the viability of the HeLa cells is over 95%. These results confirm that the UCNPs are non-toxic toward the HeLa cells. In entry III the mixture of the (**3**)-functionalized HeLa cells and the UCNPs/DOX-loaded-(**1**)-modified liposomes was subjected to NIR irradiation to induce fusion and release of UCNPs and DOX into the HeLa cells. After two days of incubation, the viability of the HeLa cells decreased to 65%, implying that, indeed, the released drug killed specifically the cancer cells. It should be noted that no cytotoxic effect on the hESC or HeLa cells was detected upon treating the cells with the UCNP/DOX-loaded-(**1**)-functionalized liposomes, in the absence of primary NIR irradiation (see Fig. S6 and accompanying discussion in the ESI†). These results reconfirm the need for NIR irradiation to induce the cleavage of the hairpin (**1**) associated with the liposomes. Only the cleaved fragment of (**1**) allows hybridization with the strand (**3**) associated with the HeLa cells and the subsequent fusion between the drug-loaded liposomes and the cancer cells. The selective fusion of the UCNPs/DOX-loaded-(**1**)-modified liposomes with the tether (**3**)-conjugated to cancer cell-specific aptamer complexes, and the selective release of DOX and cytotoxicity were further exemplified using MCF-7 breast cancer cells and MCF-10 normal epithelial breast cells (for a detailed presentation of the results see Fig. S7–S10,† and accompanying discussion). Thus, the approach presented by us provides a versatile tool for targeting the therapeutic effects of drugs towards cancer cells. It should be noted that the distribution of the fluorescent DOX in the HeLa cells could proceed by two alternative pathways: (i) fusion of the liposomes with the cell-membrane followed by release of DOX. (ii) Endocytosis of the liposome into cells followed by intracellular degradation of the liposomes which leads to the release of the drug. We find, however, the distribution of DOX in the cells proceeds on a time-scale of 25 to 30 minutes. This time-scale is very similar to the exchange rate of the contents of the fused liposomes (*cf.*Fig. 1(C)). Since the endocytosis of liposomes into cells is anticipated to be much slower (time scale of hours), the fusion pathway to release DOX is supported.

## Conclusions

The present study has introduced upconversion nanoparticle (UCNP)-loaded liposomes functionalized with photolabeled *o*-nitrobenzyl phosphate caged hairpin nucleic acid structures as functional carriers that induce photostimulated liposome–liposome fusion and liposome–cell membrane fusion processes. The UCNPs allow the NIR-irradiation of the hairpin-modified liposomes (*λ* = 980 nm) and the generation of localized UV light (*λ* = 365 nm) that deprotects and separates the hairpin structure. In the presence of nucleic acid-modified liposomes, complementary to the uncaged, hairpin-fragmented modified liposomes, inter-liposome, duplex-crosslinked structures are formed leading to the spatiotemporal fusion of the liposomes. The labeling of the separated liposomes with Tb^3+^ and 2,6-pyridinedicarboxylic acid, DPA, leads to the exchange of loads and the formation of the Tb^3+^–DPA fluorescent complex that allows the quantitative evaluation of the inter-liposome fusion efficiency (*ca.* 30%). The successful NIR photostimulated spatiotemporal fusion of liposomes by complementary nucleic acid bridges paves the way to spatio-temporal fusion of liposomes by other stimuli-responsive, reconfigurable nucleic acid crosslinkers. For example, by the modification of the two kinds of liposomes with aptamer subunits interconnection of liposomes and their fusion, through the formation of the respective ligand–aptamer complex, may be envisaged. Alternatively, the functionalization of the two kinds of liposomes with T-rich tethers and A rich tethers, respectively, is anticipated to interlink and fuse the liposomes through their crosslinking by T-A·T bridges. In addition, our study demonstrated the selective UCNP-photostimulated fusion of drug (doxorubicin)-loaded liposomes with HeLa cancer cells and the selective cytotoxicity of the drug-loaded liposomes towards the HeLa cancer cells, as compared to normal hESC cells. In this system, UCNPs/doxorubicin were loaded in the liposomes functionalized with the *o*-nitrobenzyl phosphate photo-labeled, caged, hairpin structures. The HeLa cancer cell membranes were functionalized with a nucleic acid that included the MUC-1 aptamer sequence conjugated to a nucleic strand complementary to the uncaged, fragmented, hairpin units associated with the liposomes. The aptamer units bind to the MUC-1 receptor sites associated with the HeLa cells, introducing surface functionalities for liposome–cell membrane fusion. The UCNP photostimulated-cleavage and fragmentation of the hairpin units modifying the liposomes allowed, then, the inter-hybridization of the complementary nucleic acids modifying the liposomes and cell membrane, leading to the fusion of the components, and to the release of the drug into the HeLa cells. The selective cytotoxicity of the doxorubicin-loaded liposomes towards HeLa cells is due to the aptamer guided interconnection between the liposome–cancer cell entities. The system paves the way to use liposomes loaded with other anti-cancer drugs as selective carriers for therapeutic treatment of cancer cells *via* the liposome–cancer cell fusion process. In addition, by using aptamer conjugates specific to other cancer cell biomarkers, and the appropriate engineering of the photo-responsive hairpin structures associated with the liposomes, versatile, selective targeted cytotoxicity towards different cancer cells may be envisaged.

## Conflicts of interest

The authors declare no competing financial interest.

## Supplementary Material

Supplementary information
