# Supplementary material for: Near-infrared light-activated membrane fusion for cancer cell therapeutic applications
Source: Chem Sci. 2020 May 7;11(21):5592–600. doi: 10.1039/d0sc00863j (PMC7441577; doi:10.1039/d0sc00863j)
Supplement: Supplementary file 1 [file SC-011-D0SC00863J-s001.pdf]

Electronic Supplementary Material (ESI) for Chemical Science.
This journal is © The Royal Society of Chemistry 2020

Supporting Information

Near-Infrared Light-Activated Membrane Fusion for Cancer Cell Therapeutic Applications

Fujian Huang,*,† Ruilin Duan,† Zhixin Zhou,‡ Margarita Vázquez-González,‡ Fan Xia*,† and Itamar Willner*,‡

† Engineering Research Center of Nano-Geomaterials of Ministry of Education, Faculty of Materials Science and Chemistry, China University of Geosciences, Wuhan 430074, China

‡ Institute of Chemistry, and Center for Nanoscience and Nanotechnology, The Hebrew University of Jerusalem, Jerusalem 91904, Israel

Experimental Section

Materials. 1,2-dioleoyl-sn-glycero-3-phosphocholine (DOPC), 1,2-dioleoyl-sn-glycero-3-phosphoethanolamine (DOPE), Cholesterol, 2,6-pyridinedicarboxylic acid (DPA) were purchased from Aladdin. Terbium (III) nitrate was purchased from Strem Chemicals, Inc. Doxorubicin hydrochloride (DOX) was obtained from Adamas-beta (Adamas reagent Co., Ltd). Ethylenediaminetetraacetic acid, disodium salt dehydrates (EDTA) was purchased from MACKLIN (Shanghai Macklin Biochemical Technology Co., Ltd). Unless otherwise stated, all chemicals were used without further purification. The DNA sequences functionalized with cholesterol at its 3'-end were synthesized and HPLC purified by Sangon Biotech Co., Ltd. (Shanghai, China). Cholesterol functionalized at its 5'-end oligonucleotides were synthesized and further purified by Takara Bio (Dalian, China). All DNA sequences were listed in **Table S1**. The buffer used for the experiments was Tris-HCl (20 mM Tris-HCl, 150 mM NaCl, and 1 mM EDTA; pH 7.4) for aqueous solution experiment and 1×PBS buffer (12 mM, pH=7.4 with 137 mM NaCl and 2.7 mM KCl) for cellular experiment. All the DNA concentrations were quantified by a UV absorbance spectroscopy using the absorbance of DNA at 260 nm.

The PEG amino-functionalized upconversion nanoparticles (UCNPs) composed of Yb^{3+} and Tm^{3+} (λ_{ex} = 980 nm, λ_{em} = 365 nm, 12.5 mg/mL in aqueous solution, diameter is 20 nm, zeta potential is about +5.7 mV) were purchased from Hefei Fluonano Biotech Co., Ltd. (Hefei, China).

Preparation of nucleic acid functionalized liposomes. The liposomes were prepared by the following method: 2 micromoles of DOPC, 1 micromole of DOPE and 1 micromole of cholesterol were dissolved and mixed in 2 mL chloroform in a 5 mL glass vial. The stock solution in chloroform was evaporated under a stream of N_2 to form a lipid film and further dried under high vacuum. The lipid film was rehydrated using a buffer solution (4 mL) and the suspension was vortexed. Subsequently, the suspension was repeatedly passed, using a syringe, for 21 times, through a polycarbonate membrane that includes 220 nm pores to form the liposome suspension. The final concentration of DOPC lipid in the liposome suspension was 500 μM. The mean diameter of liposomes was determined by dynamic light scattering (DLS, Malvern, Nano-ZS90). For the preparation of DPA or Tb^{3+} encapsulated liposomes, 4 mL of 2 mM Tb^{3+} and 15 mM sodium citrate, 20 mM DPA solution (prepared by 20 mM Tris-HCl buffer, 150 mM NaCl, pH 7.4) were used to hydrate the lipid films to form the liposomes at a final DOPC lipid concentration of 500 μM. Free DPA or Tb^{3+} was removed by spin-column size exclusion chromatography (GE. Healthcare Illustra Microspin S-200 HR Columns). A similar method was applied to prepare liposomes containing UCNPs and DOX. 4 mL of 0.63 mg/mL UCNPs in Tris-HCl buffer was used to hydrate the lipid films to form the liposomes containing UCNPs. We find that this sizes of the UCNPs/DOX loaded liposomes are very simila to the unloaded liposomes. As the concentration of UCNPs in the bulk solution is low, we assume that the concentration of the NPs in the liposomes is identical to their parent concentration in the bulk solution. 4 mL of 10 μM DOX in Tris-HCl buffer was used to hydrate the lipid films to form the liposomes containing DOX. The resulting liposome suspension was Pdiluted to form a liposome suspension with a final DOPC lipid concentration of 138 μM and then interacted with the corresponding cholesterol-modified nucleic acid for a time interval of 1h, bulk concentration of the nucleic acid 105 nM. The resulting nucleic acid functionalized liposomes were purified by spin-column size exclusion chromatography (GE. Healthcare

Illustra Microspin S-200 HR Columns).

Fluorescence measurements. A FS5 spectrofluorometer (Edinburgh Instruments) was used for the time-dependent fluorescence and fluorescence spectra measurements. The fluorescence emission at 490 nm of the DPA-Tb^{3+} complex was recorded using λ_{ex} = 278 nm.

Evaluation of the contents of fused liposomes (fusion efficiencies). The fluorescence of the DPA-Tb^{3+} complex in the liposome mixture subjected to fusion was measured every 60 s for 3600 s. It should be noted that leakage of contents into the solution did not lead to a signal increase as added 1 mM EDTA in reaction buffer quenches the DPA-Tb^{3+} complex fluorescence. Thus, only content exchange between liposomes is recorded as fusion. The time-dependent percentage of fluorescence increase (% F_t) was calculated using the relation: $\%F_t = (F_t - F_0) / (F_{max} - F_0)$, where F_t is the fluorescence intensity measured at time t, F_0 is the fluorescence intensity at t=0 and F_{max} is the fluorescence intensity corresponding to the complex generated upon the lysis of the mixture of liposomes using Triton X-100. The liposomes were lysed by the addition of 1 µL of 10% (w/v) Triton X-100.

(1'/1'') duplex and nucleic-acid (2) induced membrane fusion. The (**1'/1''**) duplex modified Tb^{3+}-loaded liposomes (50 µL, DOPC lipid concentration is 138 µM) were mixed with (**2**)-functionalized liposomes containing DPA (50 µL, DOPC lipid concentration is 138 µM). The time-dependent fluorescence intensities of the mixture, as a result of the Tb^{3+}-DPA complex formation upon fusion and exchange of the liposomes contents, were recorded. The change in average diameter of the liposomes was measured by dynamic light scattering (DLS, Malvern, Nano-ZS90).

Fusion of liposomes triggered by UV irradiation. (**1**) nucleic-acid-modified liposomes loaded with Tb^{3+} (50 µL, DOPC lipid concentration is 138 µM) were mixed with 50 µL (DOPC lipid concentration is 138 µM) solution that contained (**2**) nucleic acid-modified liposomes loaded with DPA. The mixed sample was irradiated with a UV lamp (LUYOR-365, China) for 3 min at 365 nm. The power of the UV source corresponded to 25 mW cm^{-2} at the irradiated sample area, determined by a power meter (LUYOR, UV 340B, China). The UV light triggered

fusion of the liposomes was followed by recording the time-dependent fluorescence changes as a result of the formation of the Tb^{3+}-DPA complex and by following the time-dependent size changes of the liposomes using dynamic light scattering (DLS).

NIR irradiation-controlled liposome fusion. A 1:1 mixture of (**1**)-modified liposomes loaded with Tb^{3+} and UCNPs and (**2**)-functionalized liposomes loaded with DPA was irradiated with a 980 nm light source (SFOLT Co., Ltd, Shanghai, China), power 1.38 W cm^{-2}, for a time-interval of 10 min. The fluorescence of the mixture before and after NIR irradiation were recorded to evaluate the fusion efficiencies. The time-dependent changes of the hydrodynamic diameter of the liposomes were determined by DLS.

Cell culture. HeLa cells and MCF-7 cells were cultured in 1640 medium (Gibco) and hESC cells were cultured in F-12 medium (Gibco), and the culture media were supplemented with 10% fetal bovine serum (Gibco) and 0.5 mg mL^{-1} penicillin-streptomycin. The MCF-10A cells were cultured in DMEM that contained horse serum, growth additive, penicillin-streptomycin (Procell Life Science &Technology Co., LTD, China). The cells were cultured at 37 °C, using a humidified 5% CO_2 incubator.

UV-light-triggered fusion of liposomes with HeLa cells. HeLa cells were first incubated with 400 μL of 105 nM hairpin nucleic-acid (**1**) PBS solution for 30 min, and the cells were washed three times with PBS. Then, 400 μL liposome L_3 (DOPC lipid concentration is 138 μM) modified with 105 nM nucleic acid (**2**) and 500 nM nucleic acid (**4**) were added to the HeLa cells. The cells were irradiated under UV light for 3 min. Following irradiation, the HeLa cells were incubated for further 30 min at 37 °C in 5% CO_2. The solution was then removed carefully and the HeLa cells were washed three times with PBS. The HeLa cells were then imaged using a Zeiss LSM 880 confocal microscope.

NIR light-controlled fusion of liposomes with HeLa cells. A PBS solution (400 μL) that included 105 nM nucleic acid (**2**) was added to the HeLa cells and incubated for 30 min at 37 °C, 5% CO_2. After incubation, the solution was carefully removed and the cells were washed with PBS 3 times. Subsequently, 400 μL PBS, that included liposomes L_1 or L_4 (DOPC lipid

concentration is 138 μM) were added to the HeLa cells. The HeLa cells were then irradiated by NIR light (980 nm) for 10 min (1.38 W cm^{-2}, 5 cycles of 2 minutes irradiation and 2 minutes break). Following irradiation, the HeLa cells were incubated for further 30 min at 37 °C in 5% CO_2. The solution was then removed carefully and the HeLa cells were washed three times with PBS. Confocal imaging was performed using a Zeiss LSM 880 confocal microscope equipped with a FemtoSecond Laser (Coherent Inc.). For the collection of the fluorescence signal of UCNPs, 980 nm FemtoSecond Laser was used to irradiate the cells.

Cell viability study. HeLa cells, hESC cells, MCF-7 cells and MCF-10A cells were seeded in a 96-well plate at a density of 5×10^3 cells/well. After 24 h culture, cells were incubated with nucleic acid (**2**) or (**5**), 105 nM for 30 min. Subsequently, the cells were washed 2-3 times with PBS. The washed cells were mixed with 400 μL the DOX and UCNPs loaded liposomes or only the UCNPs-loaded liposomes that were functionalized with the hairpin nucleic-acid (**1**). The mixture of liposomes/cells was irradiated with the 980 nm NIR laser for 10 min (1.38 W cm^{-2}, 5 cycles of 2 minutes irradiation followed by 2 minutes break). Subsequently, the cells were cultured for another two days. The incubated cells were then washed three times with PBS, and 100 μL fresh medium without fetal bovine serum, 20 μL of MTT solution (5 mg/mL) were added into each well incubated at 37 °C for another 4 h. Finally, the supernatants were aspirated, and then 150 μL of DMSO were added to each well. The absorbance intensities at 570 nm in the well were then followed using a microplate reader.

Measurement of average loading of DNA strands on one cell membrane. 500 nM of cholesterol modified DNA with FAM label in 400 μL PBS solution was added into cell culture dish (with 7.3×10^6 cells in one dish). After 30 min incubation, the fluorescence intensity of the DNA solution was measured and the concentration was calculated according to the calibration curve. The calculated concentration corresponded to 399 nM. Accordingly, the total amount of single stand DNA on cells surface corresponded to (500 nM-399 nM)×400 μL$\times6.02\times10^{23}=2.43\times10^{13}$. Thus, the number of DNA strands associated with one cell surface is: $2.43\times10^{13}/7.3\times10^6=3.33\times10^6$ strands per cell membrane.

Supporting table

Table S1: All DNA sequences used in this work

Name	Sequence
**1**	5’-AATCAACCATATCAAACTACC-**PC-linker**-TCAGATGGTAGTTTGATATGGTTGATTAGGCACGACGGA -**Chol**-3’
**1’**	5’- TCAGATGGTAGTTTGATATGGTTGATTAGGCACGACGGA-**Chol**-3’
**1’’**	5’-AATCAACCATATCAAACTACC-3’
**2**	5’-**Chol**-TCCGTCGTGCCTAATCAACCATATCAAACTACCATCTGA-3’
**3**	5’-GCAGTTGATCCTTTGGATACCCTGGTTTTTTTTTTTTTTTCCGTCGTGCCTAATCAACCATATCAAACTACCATCTGA-3’
**4**	5’-**FAM**-CGCATCTGTACTGTATTTCAC-**Chol**-3’
**5**	5’-GCAGTTGATCCTTTGGATACCCTGGTTTTTTTTTTTTTTTAGGCTATTGGTTTATCGACTATACTTAACTATTGGATTA-3’

The underline sequence corresponds to the sequence of MUC 1 aptamer.

Supporting figures: Figure S1-Figure S10

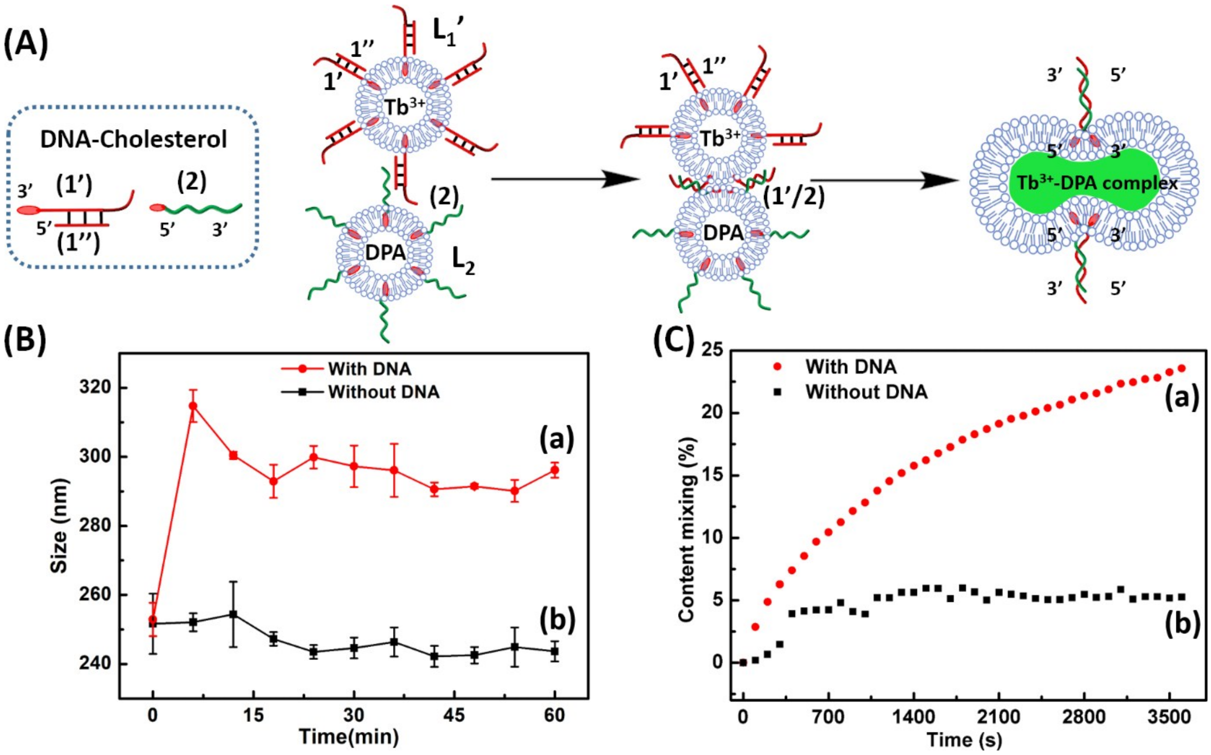

Figure S1. (A) Schematic illustration of the liposomes membrane fusion process of liposomes L_1' modified with DNA duplex (**1'/1"**) and L_2 modified with nucleic acid (**2**). The (**2**)-functionalized liposome displaces the strand (**1"**) resulting in the (**1'/2**) crosslinked liposome that leads to fusion. The fusion yields the fluorescent Tb^{3+}-DPA complex that quantifies the fusion efficiency. (B) Time-dependent size changes of the liposomes systems derived by dynamic light scattering, corresponding to: (a) A mixture of liposomes L_1' and L_2 functionalized with (**1'/1"**) and (**2**), respectively. (b) A mixture of analogue liposomes lacking the (**1'/1"**) and (**2**) nucleic acid functionalization. (C) Time-dependent percentage contents of the fused liposomes (or liposome fusion efficiency) corresponding to: (a) The interaction of liposomes L_1' and L_2. (b) Interaction of liposomes lacking the (**1'/1"**) and (**2**) nucleic acid functionalization.

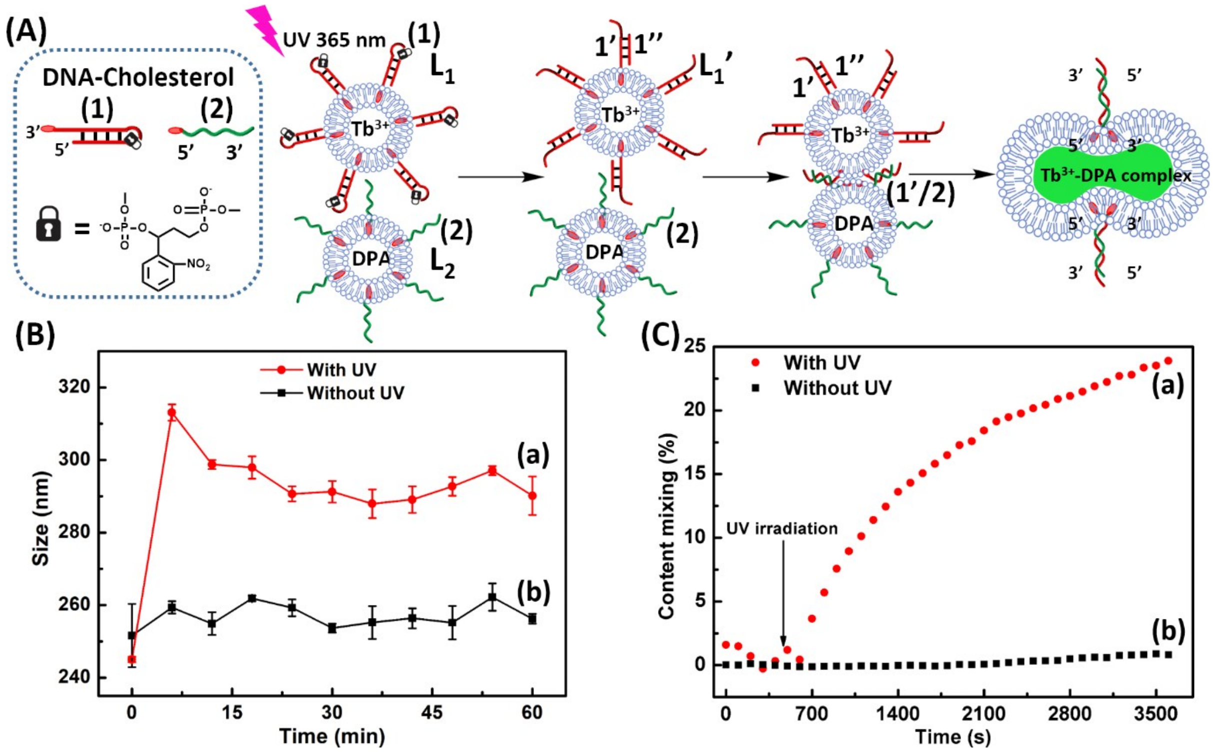

Figure S2. (A) Schematic UV irradiation-triggered fusion of liposomes L_1 modified with o-nitrophenyl phosphate locked hairpin units (**1**) and L_2 liposomes modified with nucleic acid (**2**). The liposome L_1 is loaded with Tb^{3+}-ions. Liposome L_2 is loaded with the ligand DPA. UV irradiation of the system, $\lambda = 365$ nm, deprotects the o-nitrophenyl phosphate units and fragments the hairpin structure (**1'/1"**). The (**2**)-functionalized liposome displaces the strand (**1"**) resulting in the (**1'/2**) crosslinked liposome that leads to fusion. The fusion yields the fluorescent Tb^{3+}-DPA complex and its fluorescence quantifies the fusion efficiency. (B) Time dependent size changes of the liposomes systems, derived by dynamic light scattering, corresponding to: (a) The UV-irradiated mixture of liposomes L_1 and L_2. (b) The non-irradiated mixture of L_1 and L_2. (C) Time-dependent percentage contents of the fused liposomes (or liposome fusion efficiency) corresponding to: (a) The UV-irradiated mixture of liposomes L_1 and L_2. (b) The non-irradiated mixture of the liposomes L_1 and L_2.

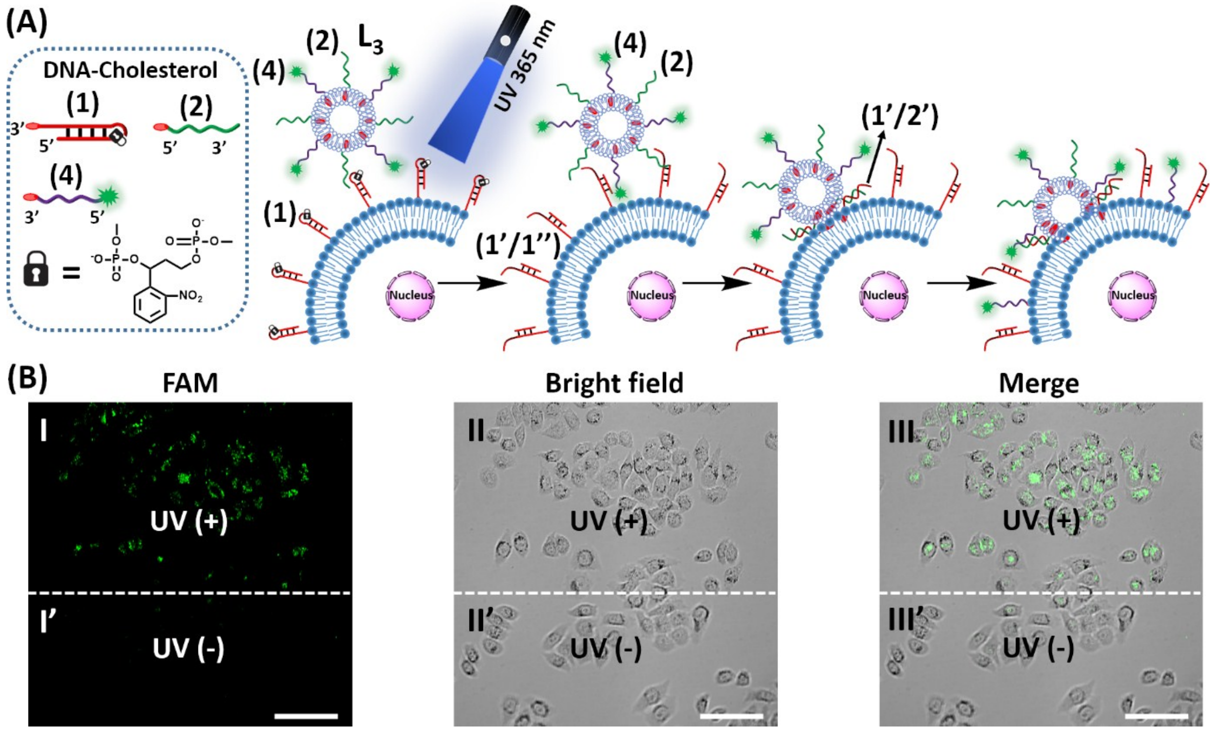

Figure S3. (A) Schematic UV irradiation-triggered fusion of the liposomes L_3 with HeLa cells modified with cholesterol-modified *o*-nitrobenzylphosphate caged hairpin nucleic acid (**1**) (average loading ca. 2.43×10^{13} (**1**) per cell). The liposomes L_3 were modified with nucleic acid (**2**) and FAM-functionalized nucleic acid tether (**4**) tethered at its 3'-end to cholesterol. The (**1**)-modified HeLa cells were selectively irradiated to uncage (**1**) and induce the fusion between the liposomes and the cells. (B) Confocal microscopy images showing the UV irradiation-triggered fusion process. The fluorescence of the FAM tethered strand, (**4**), associated with L_3 is detected on the boundaries of the HeLa Cell membranes, Panel I, implying that the liposomes were linked to the HeLa cells. The control experiment where the caged hairpin (**1**)-functionalized HeLa cells were subjected to the FAM-modified liposomes without UV irradiation did not yield the fusion of the FAM-modified liposomes with the cell membrane, Panel I'. This is consistent with the fact that the liposomes L_3 cannot bind to the caged hairpin structures associated with the HeLa cells. Panels II and III show the bright-field microscopy images and the merged images corresponding to the green channel of FAM overlayed or the bright field image of the cells, respectively. The green fluorescence of the FAM is visible on the bright-field images, indicating the fusion between the liposomes and the HeLa cells. In contrast, the bright-field images of the mixture and the merged image of the non-irradiated

mixture Panel II' and Panel III' do not show green fluorescence of the FAM-modified cell implying that no fusion of the liposomes with the HeLa cell occurred. Scale bars are 100 µm.

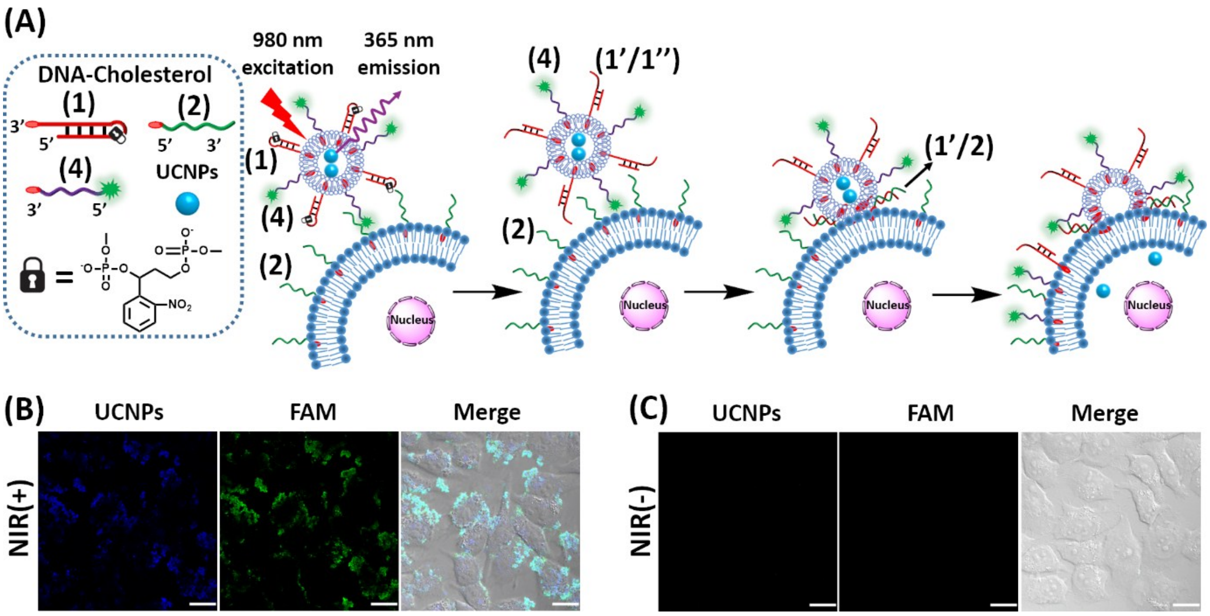

Figure S4. (A) Schematic NIR irradiation-triggered fusion of the (**1**)-hairpin and (**4**) functionalized liposomes L_4, loaded with UCNPs with HeLa cells modified the cholesterol-functionalized nucleic acid (**2**). The NIR-irradiation of the L_4/HeLa cell mixture leads to the UPNPs-stimulated cleavage of the hairpins associated with L_4. The resulting (**1'/1"**) duplexes are displaced by the tethers (**2**) linked to the HeLa cells and the contacted liposome/HeLa cells assembly results in the fusion of liposome with HeLa cells and the release of UPNPs into cytoplasm of the cells. (B) Confocal microscopy images of the cell NIR-irradiated mixture of the fused liposome/HeLa cell mixture. Blue channel is the fluorescence of the exchanged UCNPs, green channel is the fluorescence of FAM-functionalized nucleic acid tether (**4**) and the merge image demonstrates the overlap of fluorescence of the UPNPs and FAM in the fused cells. (C) Confocal microscopy images of the non-irradiated L_4/HeLa cell mixture. No fluorescence signal is observed in the cells indicating that the L_4 liposomes did not fuse with the HeLa cell in the absence of NIR irradiation**.** Scale bars are 20 µm.

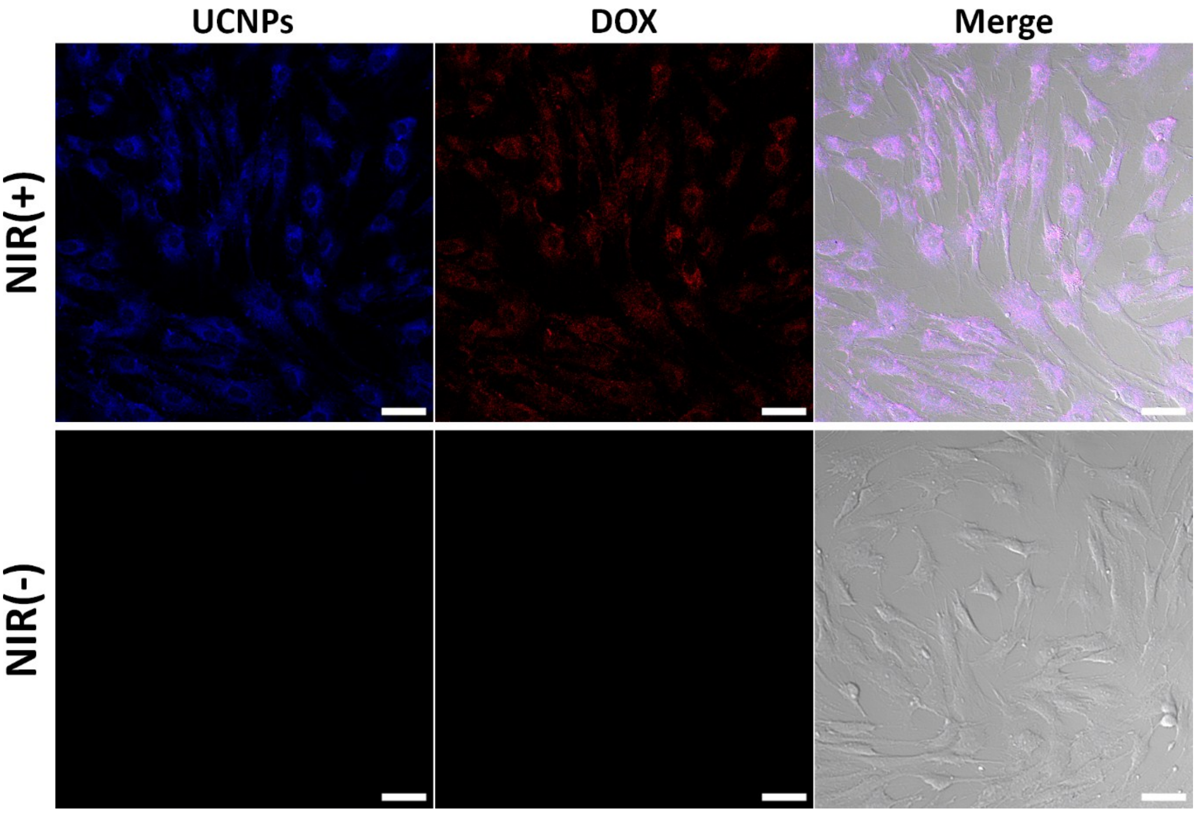

Figure S5. Confocal microscopy images of hESC normal cells treated with liposomes L_1. In this system, (**1**)-hairpin-functional liposomes L_1 were loaded with doxorubicin (DOX) and UCNPs and hESC cells were modified with the cholesterol-functionalized nucleic acid (**2**). The NIR-irradiation of the L_1/hESC cell mixture leads to the fusion of liposomes with hESC cells, which results in the release of DOX (and UCNPs) into cytoplasm (the blue and red fluorescence signals represent UCNPs and DOX fluorescence signals). No fluorescence signals are observed without NIR irradiation, indicating that L_1 liposome cannot fuse with nucleic acid (**2**) modified hESC cell without NIR irradiation. Scale bars are 20 μm.

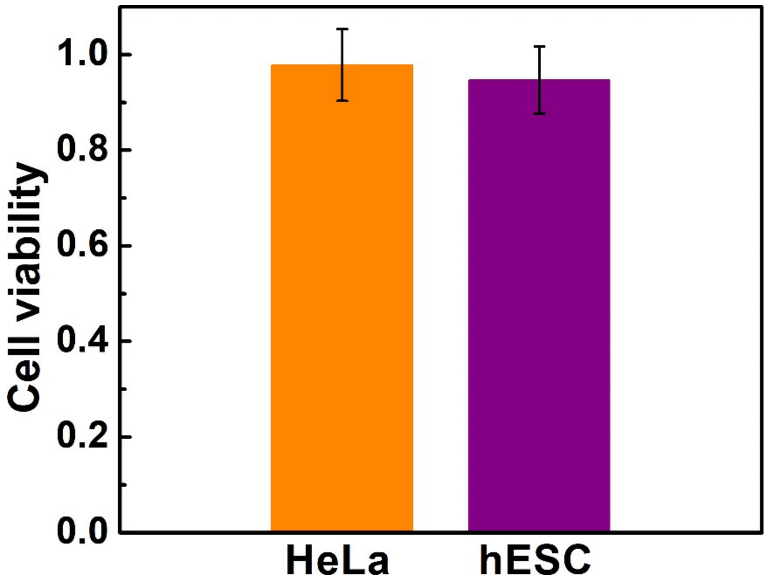

Figure S6. Cell viability of the nucleic acid (**3**) modified hESC or HeLa cells upon treating the cells with the UCNPs/DOX-loaded-(**1**)-functionalized liposomes in the absence of primary NIR irradiation. Error bars derived from N = 3 experiments.

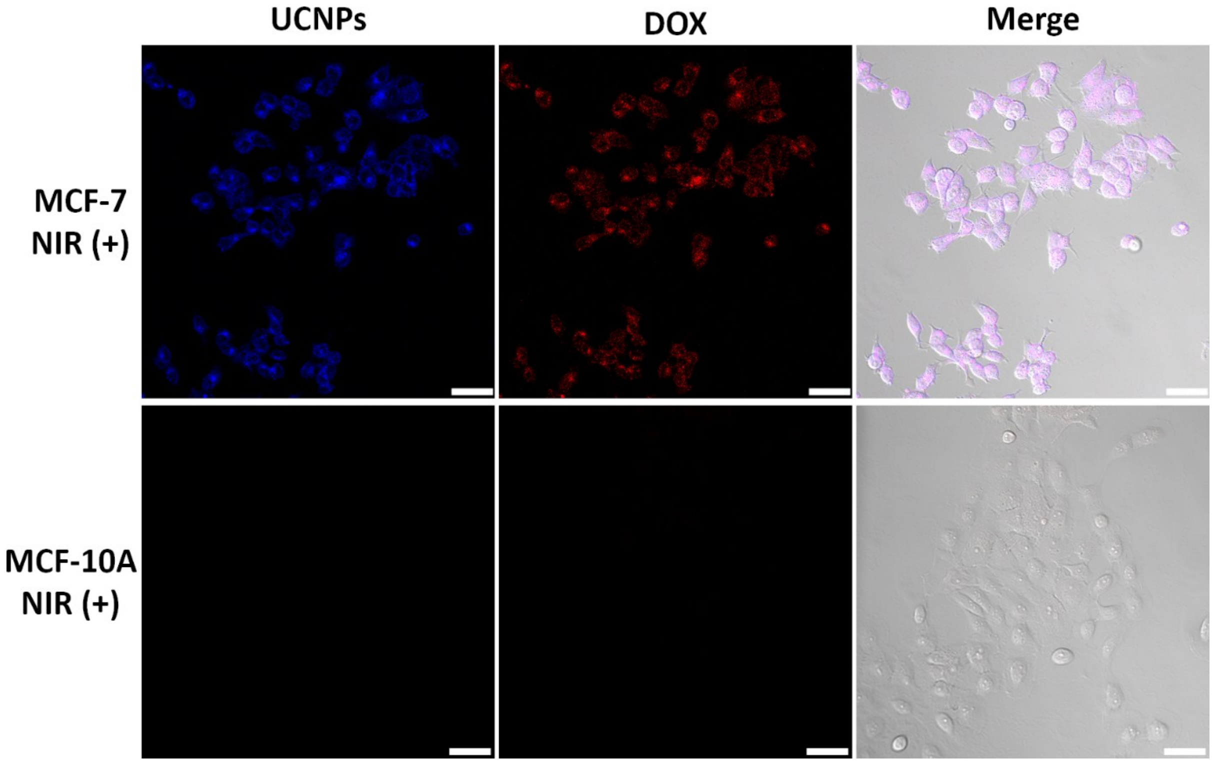

Figure S7. Confocal microscopy images corresponding to NIR-irradiated mixture of the UCNPs/DOX-loaded liposomes L_1 and the nucleic acid (**3**) treated MCF-7 and MCF-10A cells. Nucleic acid (**3**) includes the MUC-1 aptamer sequence that binds with MCF-7 cell membrane. The aptamer sequence in (**3**) cannot bind to the MCF-10 cells since their membrane lack the MUC-1 receptor site. Thus, the liposomes L_1 could fuse with MCF-7 cells but lack fusion feature with the MCF-10 cells upon NIR irradiation. Scale bars are 20 µm.

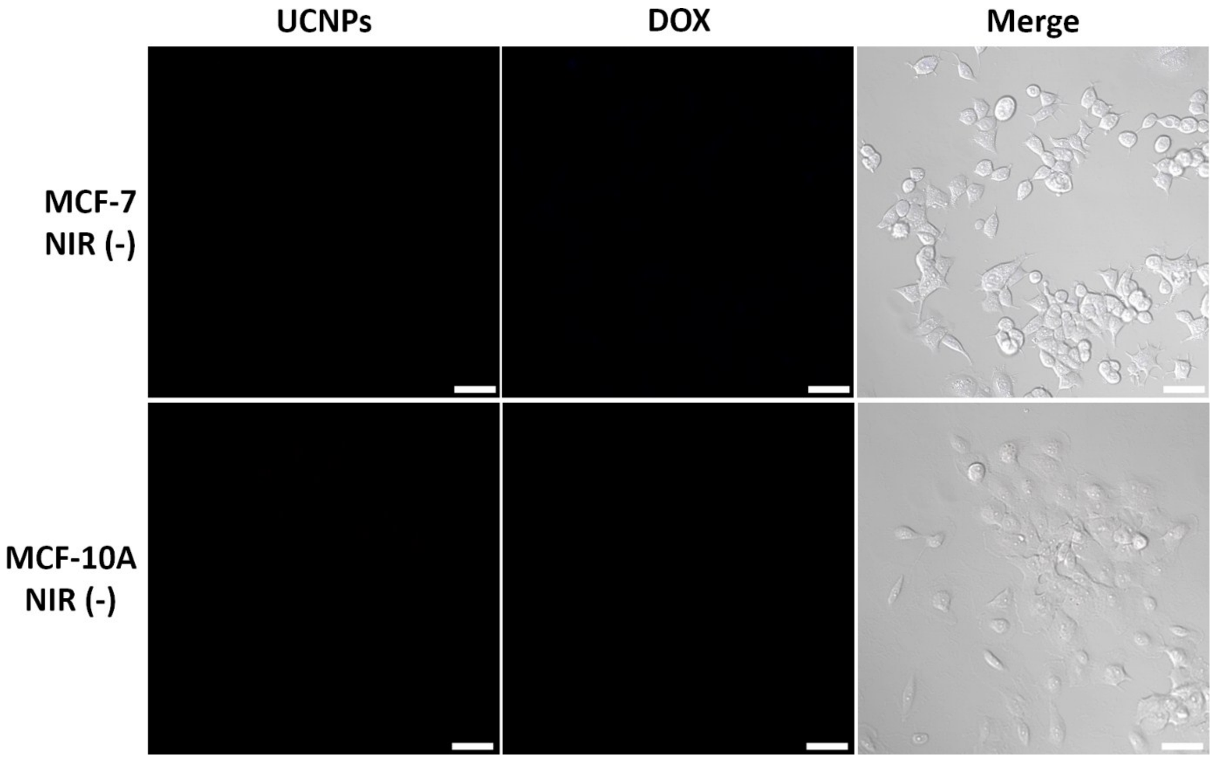

Figure S8. Confocal microscopy images corresponding to non-irradiated mixture of the UCNPs/DOX-loaded liposomes L_1 and the nucleic acid (**3**) treated MCF-7 and MCF-10A cells. Without NIR irradiation, the hairpin DNA (**1**) associate with the liposomes surface cannot be activated and no fusion between liposomes L_1 and MCF-7 (or MCF-10A) occurs. Scale bars are 20 μm.

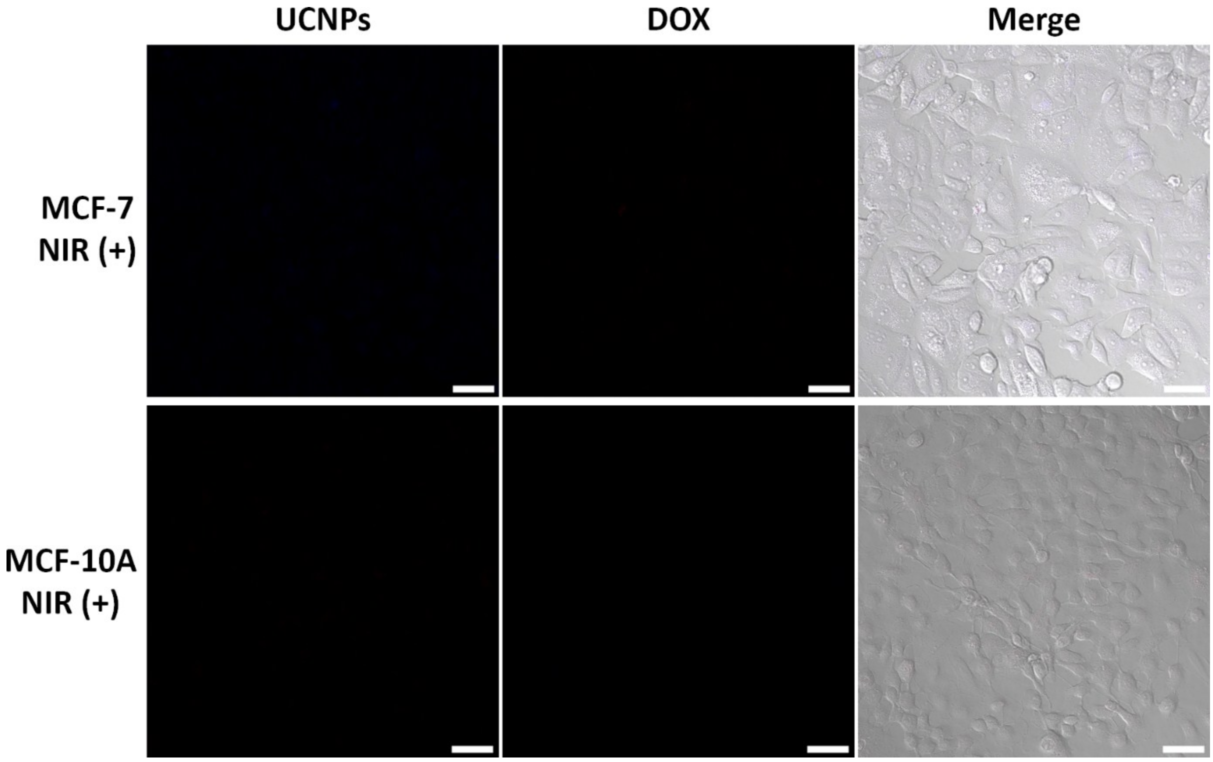

Figure S9. Confocal microscopy images corresponding to NIR-irradiated mixture of the UCNPs/DOX-loaded liposomes L_1 and the nucleic acid (**5**) treated MCF-7 and MCF-10A cells. The nucleic acid (**5**) includes the MUC-1 aptamer sequence conjugated to a random domain sequence. As the random domain sequence tether on nucleic acid (**5**) cannot hybridize with the photo-fragmented duplex (**1'/1"**), no fusion occurs. Scale bars are 20 μm.

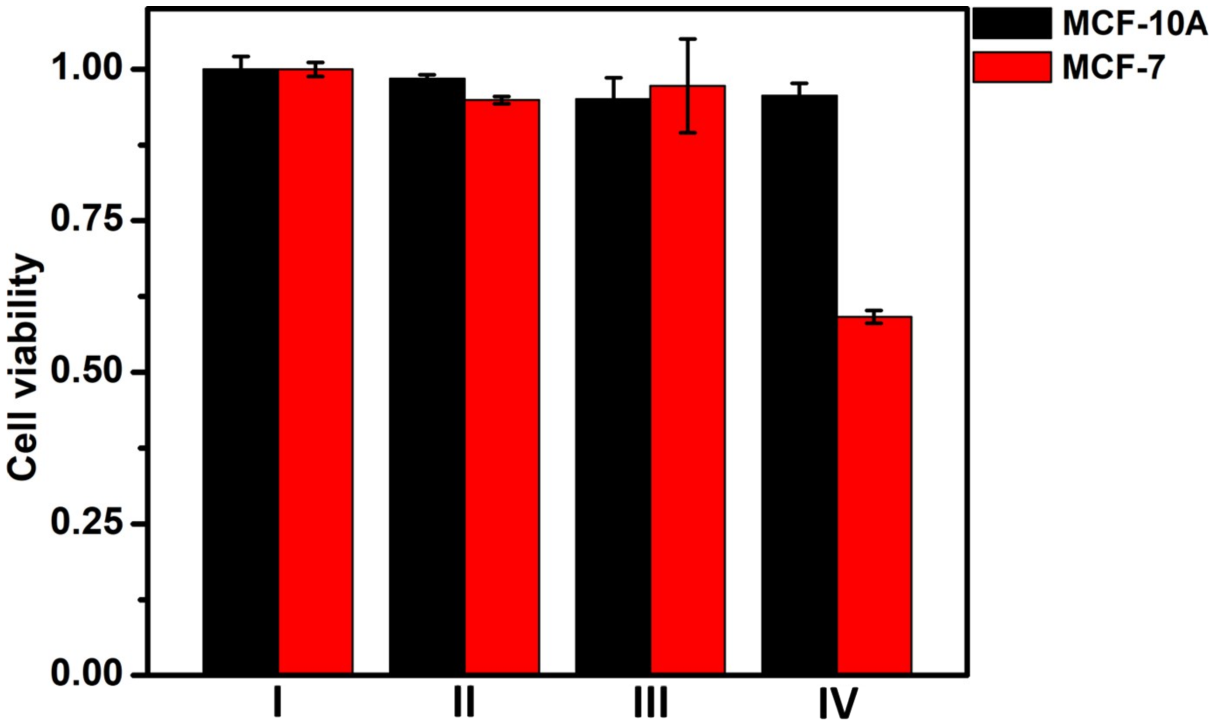

Figure S10. Cell viability of MCF-7 and MCF-10 cells treated with (**1**) functionalized UCNPs/DOX-loaded liposomes L_1 under different conditions. Entry I–non treated cells; Entry II–Cells treated with nucleic acid (**3**) and liposomes loaded with UPNPs and DOX without irradiation. Without NIR irradiation, the nucleic acid (**1**) units modifying liposomes L_1 stay intact, and thus, the L_1 liposomes cannot fuse with MCF-7 or the MCF-10 cells. Entry III–Cells treated with nucleic acid (**5**) and liposomes loaded with UPNPs and DOX upon NIR irradiation. As the random domain sequence tether on nucleic acid (**5**) cannot hybridized with the photo-fragmented duplex (**1'/1"**), the L_1 liposomes cannot fuse with MCF-7 or MCF-10 cells under NIR irradiation. Entry IV–Cells treated with nucleic acid (**3**) and liposomes loaded with UPNPs and DOX upon NIR irradiation. As the nucleic acid (**3**) can only bind to the MCF-7 cell membrane and not MCF-10 cell membrane, the L_1 liposomes fuse only with MCF-7 cells and not with the MCF-10 cells. This results in the selective cytotoxicity towards to the MCF-7 cancer cells. Error bars derived from N = 3 experiments.
